# Penile calciphylaxis

**DOI:** 10.1002/ccr3.3478

**Published:** 2020-11-06

**Authors:** Kulwant Bath, Emad Al Jaber

**Affiliations:** ^1^ University of South Alabama Medical Center Mobile AL USA

**Keywords:** dialysis, end‐stage renal disease, penile calciphylaxis

## Abstract

For small lesions, conservative management including local wound care, debridement, and adjustment of renal replacement therapy may suffice. Consulting palliative care is encouraged to help establish goals of care among patient, family, and medical team.

## Graphical abstract

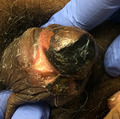

53‐year‐old man with history of hypertension, type 2 diabetes, peripheral vascular disease, and end‐stage renal disease on continuous cycling peritoneal dialysis presented to the emergency department with generalized weakness and found to have findings concerning for severe sepsis. Vital signs on presentation: T ‐max 35.7 C, BP 92/59 mm Hg, HR 97 per minutes, and RR 25 per minutes. Physical examination revealed painful blackish necrotic discoloration of the perimeatal tissues and ventral side of glans penis with no evidence of infection as shown in Figure 1. Urology was consulted and recommended supportive care with local wound care for presumed penile calciphylaxis. Biopsy was discussed but deferred due to high likelihood that the biopsy site will never heal and will lead to further decline. Laboratories revealed serum calcium 9.6 mg/dL, serum phosphorus 6.7 mg/dL, and PTH 163 pg/mL and elevated WBCs 19.2 ×10(3) cells/mcL. With the presumptive diagnosis of penile Calciphylaxis, CTA pelvis was taken, revealing extensive calcification of penile vasculature. The patient was treated with broad‐spectrum IV antibiotics, switched to hemodialysis, received adequate wound care, pain control, aggressive phosphorus control, and lower dialysate calcium with palliative care to improve quality of life. On follow up, patient is getting better with resolution of skin ulcers and improvement of pain symptoms.

**FIGURE 1:**
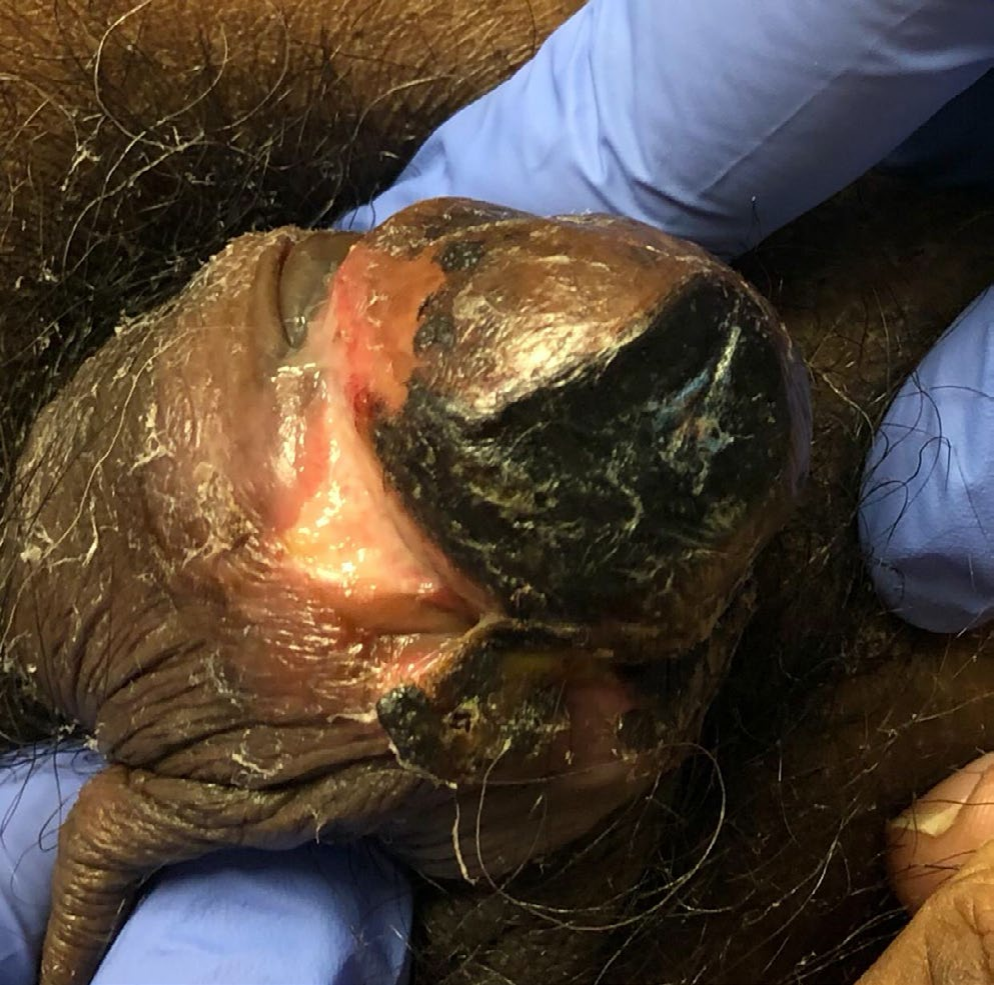
Showing blackish discoloration of the perimeatal tissues and ventral side of glans penis

Calciphylaxis or calcific uremic arteriolopathy is a syndrome characterized by calcification of vessels located in the dermis and adipose tissue. It commonly occurs in patients with end‐stage renal disease, diabetes mellitus, hypertension, and obesity.^1^ Penile involvement is an uncommon but severe manifestation. Pathologic evaluation should be used to confirm clinical suggestion. Histologic findings include medial calcification and intimal fibrosis of small to medium blood vessels without associated vasculitic change.^2^ This condition is associated with poor prognosis with high mortality rate within months of diagnosis. Goals of care discussion should be initiated early. A multidisciplinary team approach is essential, involving nephrologists, urologists, reconstructive surgeons, and palliative care with a goal to improve quality of life, given the poor prognosis and lack of evidence for management options.

## CONFLICT OF INTEREST

None declared.

## AUTHOR CONTRIBUTIONS

All authors sufficiently contributed in the intellectual content, review of literature, and analysis of data. Each author has reviewed the final version of the manuscript and approves it for publication.

## INFORMED CONSENT

Consent was taken from the patient.
